# A managers’ work engagement framework for the digital tasks

**DOI:** 10.3389/fpsyg.2023.1009459

**Published:** 2023-01-24

**Authors:** Jesus Juyumaya, Juan Pablo Torres

**Affiliations:** ^1^Escuela de Ingeniería Comercial, Facultad de Economía y Negocios, Universidad Santo Tomás, Santiago, Chile; ^2^Department of Business Administration, School of Economics and Business, University of Chile, Santiago, Chile

**Keywords:** job demands – resources model, managers, work engagement, digital managerial tasks, digital tasks

## Abstract

Unlike much research on work engagement, there is not much literature focused on managers that discuss their job demands and resources related to digital challenges in today’s organizations. Grounded in the JD-R model and considering the current digital world context, we build four research propositions and offer a work engagement framework that considers the boundary conditions of digital managerial tasks. Our conceptual framework relates the new job demands and resources to digital managerial tasks: digital adoption tasks and digital business model tasks. This conceptual article has theoretical and practical implications for organizational psychology, organizational behavior, and strategic management scholars and practitioners interested in studying managers’ work engagement and digital managerial tasks.

## 1. Introduction

Technological innovations are rapidly revolutionizing the way in which managers perceive the market and its human-machine interactions (Hsieh and Vergne, 2022). Digitalization refers to digital technologies to change a business model and provide managers with new value-producing resources (Annarelli et al., 2021). Digital businesses based on platforms, big data (BD), machine learning, robots, artificial intelligence (AI), metaverse, and algorithms are reshaping the concept of managerial work because they are altering the practices and processes of human decision-making (Sidner et al., 2005; Brougham and Haar, 2018; Sohn and Kwon, 2020). Digital technologies can take over repetitive, complicated, or heavy tasks, which leaves managers to focus more on cognitively or mentally demanding decisions (Demerouti, 2022). For instance, BD can support managers with business intelligence tools. In contrast, AI can help managers to classify information more efficiently than a team of analysts by using predictive analytics tools. The emergence of these technologies has expanded the need for research on organizational behavior and strategic management that clarifies the latest new job demands and resources that will empower managers to deal with work engagement and task performance in digital managerial tasks.

To date, there has been little agreement on the new job demands and resources related to the digital managerial tasks (Juyumaya and Torres, 2020). The job demands-resources (JD-R) model explains how resources are functional in achieving individuals’ work goals, reducing job demands and the associated physiological and psychological costs, and stimulating organizational growth, learning, and development (Bakker and Demerouti, 2017; Reina-Tamayo et al., 2017). However, the rapid technological changes in digital managerial tasks are having a serious effect on the managers’ work engagement, specifically on their vigor, dedication, and absorption (Bakker et al., 2008; Schaufeli and Bakker, 2010; Demerouti, 2022).

New job demands change managers’ mindsets about the responsibility and vigilance of these new demands. Demerouti (2022) suggests that new digital managerial tasks require individuals who pay sustained attention and react on a timely basis to a visual stimulus. Managers, who use decision-support systems, such as digital dashboards or automatically balanced scorecards, have several indicators that predict outputs automatically and over time. Yet, managers can perceive the new information displayed by the same dashboard differently (Torres et al., 2017), triggering some managers to react positively with vigor to the variation of such visual stimuli. On the other hand, others feel that the new information is overwhelming, generating a state of health impairment, such as burnout.

Furthermore, new jobs and routines demand managers who have acquired new knowledge and continuously learn (Meijerink et al., 2021). However, these changes do not occur automatically; managers face more significant pressure due to adopting new technology, increased workload, and fatigue (Andrade-Valbuena and Torres, 2018). In addition, managers need to develop new personal resources to balance new job demands with the job resources available to perform digital managerial tasks.

**Figure 1:**
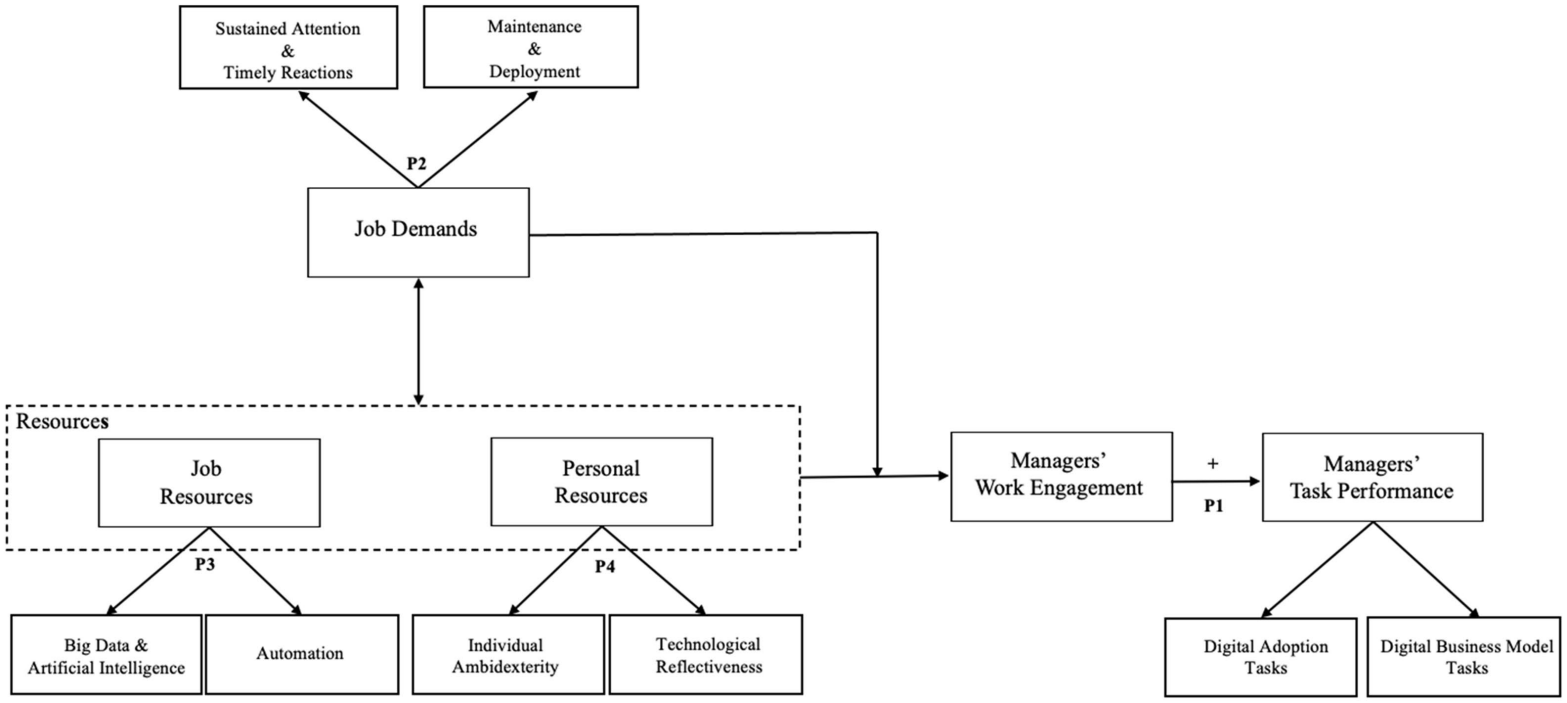
A managers’ work engagement framework for understanding digital managerial tasks. Adapted from Bakker and Demerouti (2017).

Yet the past work from organizational scholars focuses almost exclusively on the positive and negative consequences of the job resources and demands on employees’ states of mind in the workplace, with little consideration of the specific digital managerial tasks’ characteristics or the mechanisms that moderate these effects. What is missing is a coherent theoretical model of how and why new job demands and resources affect digital managerial tasks. To address this issue, we develop a theoretical framework using the JD-R model to explain how managers perceive digital managerial tasks’ job demands and resources (see Figure 1). We argue that the characteristics of digital business models have specific features that affect the manager’s work engagement. This conceptual paper attempts to answer the following questions: What new job demands and resources affect managers’ work engagement in digital managerial tasks? And what are the consequences for future research?

Knowing new job demands and resources can help managers, scholars, and policymakers create new positive strategies. In this article, we propose four research propositions. We focus on managers’ work engagement rather than employees to highlight that managers face several workplace changes that can boost or decrease task performance in digital managerial tasks. First, we offer that managers’ work engagement positively relates to task performance in digital adoption and digital business model tasks (Proposition 1). Then, we identify two new job demands (Proposition 2), two new job resources (Proposition 3), and two new personal resources (Proposition 4), which are associated with the manager’s digital managerial tasks. These three research propositions lay the foundations for further empirical explorations.

This paper will benefit practicing managers and academic scholars in research and development (R&D), human resources, innovation management, and, more broadly, organizational behavior and strategic management researchers. We first briefly describe the JD-R model and its extensions to explain digital managerial tasks. We then present several novel research propositions as to how combinations of different job demands and resources influence different aspects of digital task performance through their effects on managers’ engagement. Finally, we describe the implications of our theoretical model for both theory and practice and discuss streams for future studies.

## 2. Work engagement in digital managerial tasks

Traditionally, the JD-R model has been used to predict some positive organizational effects of traditional jobs on employees, such as motivation, organizational commitment, work engagement, and task performance (Schaufeli and Salanova, 2007; Christian et al., 2011; Juyumaya, 2022), juxtaposed to adverse organizational outcomes, like stress, exhaustion, burnout, sickness, absenteeism (Schaufeli et al., 2008; Crawford et al., 2010; Breevaart et al., 2015). JD-R model analyzes job characteristics into two categories: job demands and job resources. Job demands refer to the physical, psychological, social, or organizational aspects that require physical and psychological effort and are related to physiological and psychological costs, such as work pressure and emotionally demanding interactions with board members, strategic partners, and clients (El-Kot and Burke, 2010). On the other hand, job resources refer to the physical, psychological, social, or organizational aspects of the job that are (a) functional in achieving work goals; (b) reducing job demands and the associated physiological and psychological costs; or (c) stimulating personal growth, learning, and development (Bakker et al., 2011).

Figure 1 outlines the four propositions within the work engagement framework for digital managerial tasks. It is based on the JD-R model (Bakker and Demerouti, 2017). This framework explores a number of four research propositions about the role of digital managerial tasks within the work engagement framework. It suggests how new job demands and resources could be further explored concerning digital managerial tasks. The framework’s left side depicts new job demands (sustained attention and timely reactions and maintenance and deployment), new job resources (big data and artificial intelligence, and automation), and new personal resources (technological reflectiveness and individual ambidexterity). The right half of the framework illustrates the results of managers’ work engagement. It concerns the impact of job demands and resources on task performance (digital adoption tasks and digital business model tasks), following an individual level of analysis.

## 3. Effects of digital managerial tasks on managers’ work engagement

Creating and adopting new digital business models is a permanent task for managers that involves new job demands and new resources in digital managerial tasks. For example, these digital managerial tasks allow managers to understand how digital users and customers adapt their products and services. We discuss two digital managerial tasks embedded in the work engagement framework for digital managerial tasks: (1) digital adoption and (2) digital business models.

### 3.1. Digital adoption tasks

Digital adoption occurs when a manager acquires managing technology and successfully carries out digital business objectives. Managers that manage digital adoption can (a) understand the potential of digital resources, (b) accept and utilize such resources, and (c) deploy technology to optimize processes and grow innovation. The increased standardization of tasks replaces routine cognitive skills, and automation causes a decline in the active use of skills and an increase in monitoring tasks (Parker and Grote, 2022). Managers need to collect data from different sources to enhance their task performance. However, most captured data are of value only when combined with other data in a specific context. Information silos vary widely across disciplines. The greatest challenge in many cases is to dismantle information silos and incorporate these islands of information. Zhan and Tan (2020) propose an analytic framework for collecting extensive data to enhance performance that comprises five main stages. Stage 1: data capture and management. Stage 2: data cleaning and integration. Stage 3: data analytics. Stage 4: competence set analysis-deduction graph. Stage 5: information interpretation and decision-making.

Digital adoption is a crucial aspect of these five main stages. For instance, in Stage 1, organizations must determine the data sources to create as much value as possible. Therefore, the company needs managers to assume tasks to be able to define the data to be captured and manage such data. In Stage 2, managers must classify and transfer data from different sources. Stage 3 must apply analytics and mining algorithms to identify competence-set information. Then, they use a deduction graph for competence-set analysis in Stage 4. Finally, in Stage 5, they generate a competence network and results for competence-set analysis and decision-making. Managers’ vigor, dedication, and absorption reduce the negative influence of complex job demands, such as sustained attention and timely reaction and deployment and maintenance. As a result, engaged managers are probably more likely to accept, integrate, use and promote new digital technology in their companies (Demerouti, 2022).

### 3.2. Digital business model tasks

Technological innovation does not guarantee business success. Often, products and services can be easily copied, whereas business model innovation can provide more sustainable market success. Consequently, new product or service development efforts should be coupled with a business model that defines go-to-market and value-capturing strategies. Business models can change industries and drive exceptional growth (Johnson et al., 2008). Insight into these effects has caused business models to garner increasing attention in practice and research. The digital and platform economy’s innovative business models are built on software and internet-based technologies such as big data and artificial intelligence (BDAI).

The unprecedented development of information and communication technology (ICT) has led to a phenomenon known as digitalization. Business models are adopted, especially by young enterprises. In this scenario, classical business models funded predominantly on physical activities are disrupted and shifting toward digitalization. Therefore, digitalization affects an organization’s business model and all segments of society, including new relationships and interactions with organizations. Digitalization has consequences for all industries (Buyukozkan and Gocer, 2018).

Although many activities may be digitalized, talented professionals remain a strategic resource. Human capabilities are essential in helping and creating a business model in this new digital context (Sivathanu and Pillai, 2018). The human factor is also vital to a firm’s performance (Xanthopoulou et al., 2009b; Gunasekaran et al., 2017; Afshan and Motwani, 2018). The requirement for new human capabilities challenges an organization’s development. For instance, how can firms support data scientists’ formation? What is the role of the organization following job losses resulting from digitalization?

Croll and Yoskovitz (2013) propose five tasks that managers should implement to build a digital business model: (1) create the acquisition channel, (2) define the selling tactic, (3) formulate the revenue source, (4) build the digital product type, and (5) agree with the form of delivery. These tasks are part of the digital business model tasks. Table 1 shows digital managerial tasks, questions, and tactics.

**Table 1:** Linking digital business model tasks with the new job demands and resources.

Digital managerial tasks	Main questions	Tactics	Job demands	Job resources	Personal resources
Acquisition channel	How do the digital visitors, users, or customers determine the firm?	Paid advertising; search engine management; artificial virality app ecosystem	Sustained attention and timely reactions; maintenance and deployment	Big data, predictive analytics, and artificial intelligence; automation	Technological reflectiveness; individual ambidexterity
Selling tactic	How does the firm convince visitors, users, or customers to become paying customers?	Discounts free trial freemium	Sustained attention and timely reactions; maintenance and deployment	Big data, predictive analytics, and artificial intelligence; automation	Technological reflectiveness; individual ambidexterity
Revenue model	How does the firm extract money from its visitors, users, or customers?	Subscription consumption charges advertising clicks	Maintenance and deployment	Big data, predictive analytics, and artificial intelligence	Individual ambidexterity
Product type	What does the firm offer in return for the revenue?	Software platform marketplace	Sustained attention and timely reactions	Automation	Technological reflectiveness
Delivery model	How does the product get to customers?	Digital delivery hosted service	Sustained attention and timely reactions	Automation	Technological reflectiveness

In the current digital world, new business models and value creation are the mechanisms that allow new job demands and resources to emerge and, consequently, increase managers’ work engagement and task performance. These new job demands and resources are part of a new-thinking business model focused on managers’ work engagement. For instance, for all the digital managerial tasks presented in Table 1, BDAI not only promotes more information about customers (acquisition channel and selling tactic) but also, most importantly, increases the accuracy of this information (Buyukozkan and Gocer, 2018) thanks to the adoption of innovative technology (revenue model). Consequently, BDAI entails smart connections between organizations and their customers. Additionally, customer integration continues to be a vital subject related to an organization’s performance (product type and delivery framework; Al Halbusi et al., 2022) because managers’ work engagement involves the three dimensions of the construct: vigor, dedication, and absorption, especially concerning its cognitive dimensions: (1) vigor: energy and mental resistance; and (2) absorption: visual attention and concentration. Managers must use their energy, mental resistance, visual attention, and concentration to respond to these digital business model tasks.

This framework highlights the need for organizations to consider that managers’ work engagement is critical to success and needs to be managed and developed. Managers’ work engagement increases task performance and generates more resources to face new job demands. Furthermore, managers’ work engagement is positively related to managers’ task performance through job-fit (Venkatesh et al., 2003). Managers can create a better digital business model and adopt new digital technology for their companies when engaged. Consequently,

*Proposition 1*: Managers’ work engagement is positively related to task performance in digital adoption tasks and digital business model tasks.

## 4. Consequences of digital managerial tasks on the JD-R model

Whereas meaningful job demands and resources can be found in almost every top management team, some job demands and resources are unique and related to the digital business context. For instance, physical demands, such as visiting customers across cities, are still essential for traditional real state managers. In contrast, cognitive demands are much more relevant for chief scientist officers in fintech companies who are dealing with the problem of finding the optimal portfolio of real estate assets in a digital platform.

JD-R model proposes two effects in which demands and resources may have a combined effect on managers’ work engagement and indirectly increase their task performance (Bakker and Demerouti, 2017). Firstly, job resources buffer the impact of job demands on the strain. Research has shown that job resources, such as social support, autonomy, performance feedback, and development opportunities, can face managerial demands, including the manager’s burnout (Xanthopoulou et al., 2009a). Therefore, managers with many personal resources can cope better with their job demands. Secondly, job demands amplify the impact of job resources on work engagement (Bakker and Demerouti, 2017). Bakker and Albrecht (2018) suggest that job resources become salient and have the most decisive positive impact on work engagement when job demands are high. When a manager is confronted with challenging job demands, job and personal resources become valuable and foster vigor, dedication, and absorption in the tasks. It includes the investment of cognitive, emotional, and behavioral energies in work, dramatically impacting job satisfaction and performance (Kahn, 1990; Rich et al., 2010).

Based on Bakker and Demerouti (2017), job demands and resources can evoke two separated processes in digital managerial tasks: (1) an energetic process of wearing out in which high job demands exhaust managers’ cognitive resources and may therefore lead to burnout and eventually ill health; and (2) a motivational process in which job resources boost managers’ work engagement. These characteristics do not differ from previous descriptions of employees’ work; nevertheless, digital work also forces managers to reflect on adopting technology and how these technologies will positively impact task performance (Andrade-Valbuena and Torres, 2018). The early focus of studies using the JD-R model centered primarily on front-line employees, nonmanagerial roles employees, and first-line managers. With our framework, we emphasize the analysis on the managers (middle managers and top managers). Further, past research study job attributes focused on inputs, repetitive tasks, traditional skills, clearly defined tasks, siloed metrics, and slow and methodical work (e.g., Luoma, 2000). While our conceptual study discusses the framework primarily focused on outputs, *ad hoc* activities, e-skills, unclear tasks, integrated functions, KPIs, and fast, agile, and efficient job attributes (e.g., Hofmann and Rusch, 2017).

Empirical evidence suggests that managers experiencing technological failures [e.g., chief technology officers (CTOs)] lead their teams in significant work challenges, such as achieving better standardization and continuously removing dysfunctional routines (Say and Vasudeva, 2020). Firms pursuing an aggressive technology strategy in industries where technology is a critical contingency, such as high R&D spending, encourage CTOs to concentrate entirely on the success of new innovative projects through a higher compensation scheme (Medcof and Lee, 2017). Hence, engaged managers who are highly active, enthusiastic about, and often fully immersed in their work should enhance organizational routines associated with digital managerial tasks. Table 2 compares traditional and digital managerial tasks using the fundamental characteristics of the JD-R framework.

**Table 2:** Traditional vs. digital managerial tasks in the JD-R model.

Characteristics	Job demands	Authors	Job resources	Authors	Personal resources	Authors	Positive results	Authors	Negative results	Authors
Traditional Tasks	Workload; emotional demand; work-home conflict; interpersonal violence	Demerouti et al. (2001), Schaufeli and Salanova (2007)	Autonomy; transformational leadership; skill variety; group support	Juyumaya and Torres (2023), Holman and Axtell (2015), Tanveer et al. (2021)	Optimism; self-efficacy; resilience; self-esteem	Bakker et al. (2011), Mäkikangas et al. (2013)	Work engagement; commitment	Hakanen et al. (2005), Jong and Ford (2016)	Exhaustion, burnout	Demerouti et al. (2004), Bakker and Costa (2014)
Digital managerial tasks	Sustained attention and timely reactions; Maintenance and deployment	Demerouti (2022), Parker and Grote (2022)	BDAI; Automation	Hazen et al. (2012), Demerouti (2022)	Technological reflectiveness; Individual ambidexterity	Schweitzer et al. (2015), Andrade-Valbuena and Torres (2018), Mom et al. (2009), Torres et al. (2015)	Occupational health; Well-being	Demerouti (2022), Parker and Grote (2022)	Impaired health; Loss of situational awareness	Demerouti (2022), Parker and Grote (2022)

### 4.1. New job demands

#### 4.1.1. Sustained attention and timely reaction

Digital transformation has become mandatory for all firms and businesses. The new context is increased job demands as byproducts of technology introduction or an intrusion into private life facilitated by technology. For instance, evidence shows that the COVID-19 pandemic increases leader workload and work–family conflict. Technology can influence managers’ well-being and make them more exhausted and less engaged. Digitalization can provide opportunities and constraints for managers’ well-being and health and for managers’ abilities to participate and thus co-shape the developments they are affected (Wilkinson et al., 2021).

Demerouti (2022) states that technology takes over the treatment, manipulation, or assembly of smaller objects, parts, or information; the human operator must work with more cognitively or mentally demanding features that require processing a significant amount of data. Additionally, workload variation due to automation, from underload to rapid overload (Parker and Grote, 2022), requires managers to react on a timely basis. As Bainbridge (1983) noted, it is impossible for even a highly motivated human being to maintain adequate visual attention toward a source of information. In cognitive neuroscience, visual attention refers to cognitive operations that mediate the selection of relevant information and filter irrelevant information from cluttered graphic scenes. Currently, managers face more visual attention and timely reaction demands, such as following the latest, complex, balanced scorecard or other business intelligence tools. Job demands based on physical strength have shifted to the demand for timely reactions and sustained attention.

A large amount of data demands that managers pay visual attention and react opportunely. Managers need to respond quickly to various stimuli and then manage the information to deploy a creative/innovative solution to diverse business problems, such as reading algorithm black boxes, giving timely responses to customers and clients, or managing the relationships between the company and stakeholders/shareholders because of the latter’s requests through various digital platforms.

#### 4.1.2. Maintenance and deployment

We argue that job demands change as managers take over maintenance and deployment tasks. Under certain conditions, firms can favor employment structures with less skilled routine work but more highly technical professional work related to control, planning, maintenance, and deployment. Combining big data and artificial intelligence with other technologies creates additional capabilities for digitally transformed firms. These capabilities can develop economies of scale and scope for the organization. Maintenance tasks are required to ensure optimal operating systems. Paradoxically, technology has supported and reduced specific tasks but has introduced new workloads associated with maintaining software, robots, and platforms that provide management control and monitoring. Managers involved in digital managerial tasks increase the job demands of surveillance and performance monitoring (Parker and Grote, 2022) associated with these digital environments.

On the other hand, managers face deployment tasks (Parker and Grote, 2022). Deployment is where data mining yields results that impact task performance. The Cross-Industry Standard Data Mining Process, known as CRISP-DM, is an open standard process framework that indicates approaches used by data mining practitioners. According to CRISP-DM (Shearer, 2000), managers use data information to improve the way to do business or generate new forms of income through a value proposition embedded in a digital business model. Managers must continuously acquire new knowledge to validate, sort, and analyze big data to make strategic decisions (Meijerink et al., 2021). These new tasks require specific know-how and skills related to exploration and exploitation.

In digital managerial tasks, the purpose of human engagement with machines has a dual process, where managers or highly skilled employees enhance their decision-making process by augmenting their managerial capabilities. Individual ambidexterity can be an appropriate personal resource for managers to help complete deployment tasks (Torres et al., 2015). However, low-qualified employees are reduced to only those aspects where robotic machines still lack capabilities. For example, the gig worker is controlled by digital platforms and algorithms. Still, gig managers can make critical decisions across different countries with detailed information in real-time (*cf.* Ravenelle, 2019).

Managers see their autonomy and job complexity augmented as there is a shift toward valuing “mental work” over “manual work” (Sennett, 2008). Human engagement is significantly mediated or replaced by mechanization and algorithmic control. The work process is highly structured and overdetermined; standardization is vital for efficiency and consistency. Most deployment and maintenance tasks are highly structured, involving significant time and cognitive stress. The effect of a job resource is dependent upon the micro-context. For example, job demands or other job resources are determined by the level and nature of job demands (van Veldhoven et al., 2020). In digital managerial tasks, job demands and resources are characterized by the micro-tasks described before (e.g., sustained attention, timely reaction, and maintenance and deployment). Therefore, the managers’ work engagement proposes that job demands can increase or decrease managers’ work engagement. This could be further explored for a more nuanced perspective of job demands. Thus,

*Proposition 2*: Sustained attention, timely reaction, and maintenance and deployment are job demands in digital managerial tasks.

### 4.2. New job resources

#### 4.2.1. Big data and artificial intelligence

Our proposed framework groups big data and artificial intelligence (BDAI) with several job resources, such as big data, predictive analysis, machine learning, and other computational algorithms. The traditional JD-R model considers resources in terms of physical capital, human capital, technological capital, and tangible or intangible capital (Gunasekaran et al., 2016). We consider big data a job resource because this body of knowledge is responsible for analyzing and extracting data from large sets that are too complex to be dealt with by traditional data-processing tools (Chen and Zhang, 2014). Big data resources include capturing, storing, analyzing, searching, sharing, transferring, visualization, querying, and updating information privacy and data sources (McAfee and Brynjolfsson, 2012). New interpretations of the JD-R model consider AI and automation because they enable a firm to deal with tasks that are difficult for humans because of the complexity of the operations and procedures (Gunasekaran et al., 2016).

Despite the numerous advancements of BDAI, a large part of the existing literature considers them to be domains related only to a firm’s processes and infrastructures (Chen and Zhang, 2014), while a few articles have put attention on the role of managerial cognition in facing new job demands based on BDAI (Caputo et al., 2019). An organization must develop BDAI acceptance and assimilation capabilities through BDAI routinization (Hazen et al., 2012). BDAI requires as much theoretical knowledge as it does a wide array of quantitative skills. Our framework supports the idea that BDAI is a job resource that can increase managers’ work engagement because it involves a cognitive process of knowledge assimilation.

Managers are more likely to feel more engaged when their company’s system and infrastructure have platforms and BDAI infrastructure because they need to address rapidly changing environments that integrate building and reconfigure internal and external data (Hazen et al., 2012). From a dynamic managerial capability perspective (Helfat and Peteraf, 2015), an organization needs to develop BDAI acceptance and assimilation capabilities through the mediating construct of BDAI routinization. Routinization is the permanent adjustment of an organization’s governance system to incorporate technology. Hazen et al. (2012) argue that routinization is the second stage of a threefold process: acceptance, routinization, and assimilation. Organizations must accept, routinize, and assimilate technologies to generate BDAI assimilation (Gunasekaran et al., 2016).

We argue that companies with BDAI resources could increase work engagement because managers perceive such resources to be relevant in dealing with new job demands. Caputo et al. (2019) state that it is necessary to accept, establish routines, and assimilate new technologies to obtain benefits. In this regard, in the BDAI process, the first stage is acceptance, routinization, and finally, assimilation. BDAI resources also include technology and market surveillance processes, enabling managers to understand the current and latest trends in industries and markets. BDAI assimilation empowers a manager to think about the impact of a technological product on its users and society in general because BDAI environments stimulate this type of thinking (Gunasekaran et al., 2016).

#### 4.2.2. Automation

Automation is a new technology by which a process or procedure is performed without direct human assistance (Parker and Grote, 2022). For instance, managers can actively change the design of their jobs by choosing tasks, negotiating different job content, and assigning meaning to their tasks or jobs. The availability of healthy-designed employment and working conditions facilitates manager motivation and reduces stress (Demerouti, 2022).

Automation uses various equipment operating control systems, such as machinery, processes in factories, boilers, heat-treating ovens, switching on telephone networks, steering and stabilizing ships, aircraft, and other applications and vehicles with minimal or reduced human intervention. Following the analysis of Demerouti (2022), automation can contribute to work-related health if (a) they are designed to support the work of individuals, (b) individuals are in control and can craft their use, (c) job resources are maximized, and job demands are affordable, (d) economic growth is shared among stakeholders, including managers, and (e) authorities protect managers and employees.

Job crafting represents a bottom-up adjustment of the tasks and characteristics of the job to fit one’s preferences and find meaning in the position. According to the JD-R model, managers can craft their job by expanding (i.e., seeking resources and challenges) or reducing their scope (i.e., diminishing or optimizing demands; Demerouti, 2022). Automation and job crafting may be an excellent combination of resources to increase managers’ work engagement in digital managerial tasks. Accordingly, automation reduces cognitive and physical goals because it supports a manager’s digital managerial tasks. To turn BDAI and automation into job resources, we need to analyze the nature and amount of the job resource (Van Veldhoven et al., 2020), as well as the way the job resource is valued by the managers as regards the task goal under consideration. Then,

*Proposition 3*: Big data and artificial intelligence, and automation are job resources related to digital managerial tasks.

### 4.3. New personal resources

#### 4.3.1. Technological reflectiveness

We recognize a unique personal resource called technological reflectiveness, an individual’s tendency to think about the impact of a technical product on different stakeholders, such as employees, customers, and shareholders (Schweitzer et al., 2015). Technological reflectiveness draws on theories on reflection and reflexivity (Trapnell and Campbell, 1999). The managers’ work engagement framework proposes that technological reflectiveness is a personal resource that can increase work engagement in the digital workplace characterized by digital managerial tasks. In fact, managers are more likely to feel more engaged when they have the individual tendency of technological reflectiveness (Andrade-Valbuena and Torres, 2018).

The literature about technological reflectiveness aims to understand which individuals are effective contributors to technical innovations because they take society into account. In light of companies’ R&D areas increasing endeavors to open up the innovation process and seize the abilities and skills of external sources in the innovation process (Gassmann, 2006), the measure of technological reflectiveness can be used to recruit externals with high technology reflectiveness scores to contribute to the innovation process. Despite the significance of technological reflectiveness, no previous studies link this resource to work engagement. Technological reflectiveness can supplement other job and personal resources, such as autonomy, supervisory encouragement, team support, and managerial self-efficacy (Tierney and Farmer, 2002), to achieve managers’ work engagement.

A creative work environment may enhance a manager’s feeling of control and resiliency, motivating them intrinsically (Amabile, 1997), a prerequisite for work engagement (Xanthopoulou et al., 2013). Managers in digital managerial tasks must reflect deeply on how new technologies impact technical solutions in the firm, market, and society (Heavin and Power, 2018). Technological reflectiveness is significantly related to the individual’s willingness to promote new technologies (Andrade-Valbuena and Torres, 2018). Hence, technological reflectiveness can be a crucial resource in achieving managers’ work engagement because thinking about the impact of a technical product on its users and society can be critical in digital environments (Venkatesh et al., 2003).

#### 4.3.2. Individual ambidexterity

According to Mom et al. (2009), individual ambidexterity is a managerial orientation toward combining exploration-and exploitation-related activities within a particular timeframe. In the last decade, individual ambidexterity has become more concentrated on how leaders act ambidextrously to increase individual or group performance (Tushman and O’Reilly, 1996).

The digital JD-R approach offers some arguments to support the idea that individual ambidexterity can be a personal resource that can boost managers’ work engagement (McCauley et al., 1994). Managers prepare strategic plans that involve short-and long-term investments (e.g., capital expenditures). Such initiatives must be approved annually by the board of directors. Strategy formulation requires significant resource mobilization, coordination, and integration to maintain exploitation and exploration activities (Gibson and Birkinshaw, 2004).

Managers are involved in several tasks, such as information sharing and knowledge processing, that require balancing short-and long-term tensions (Rosenkopf and Nerkar, 2001). Moreover, managers can access the most valuable and diverse information to avoid separating explorative and exploitative behaviors (Sidhu et al., 2004).

Ambidextrous managers engage in complex cognitive processes such as integrative or paradoxical thinking to reconcile the tensions that may emerge in their pursuit of a range of different opportunities, goals, and needs that seem to conflict in terms of time horizon, risk profile, relationship to the current strategy, and managerial responsibilities (Audia et al., 2000). Ambidextrous managers are skilled at not stressing the polarity of seemingly conflicting opportunities, goals, and needs (March, 1991). They are motivated to develop creative solutions that combine all aspects by emphasizing their interrelatedness.

Another commonality is that ambidextrous managers are skilled and motivated to engage in a vast repertoire of different or opposing activities and roles, such as conducting routine and nonroutine activities, leadership roles, and performing entrepreneurial and creative tasks (Adler et al., 1999). Further, managers can shift attention quickly between such different behaviors and functions, depending on the situation and the broader interests of the organization (Bernett, 2008). In this sense, ambidextrous managers have been called multitaskers and generalists rather than specialists (Mom et al., 2015).

Previous studies indicate that ambidextrous managers have the skills and motivation to engage in learning activities, such as reliability enhancement, and increase learning activities to refine and refresh their knowledge and skills (Torres et al., 2015). They build internal linkages to cooperate, combine efforts with others, and develop and maintain rather large, dense personal networks to share knowledge and information (Van Wijk et al., 2008).

We believe that individual ambidexterity can be a critical personal resource for managers that fosters explorative and exploitative behavior in managers by increasing or reducing the flexibility of desirable behavior (Mom et al., 2009). Considering the nature of digital managerial tasks, these individual tendencies of technological reflectiveness and individual ambidexterity can be essential personal resources for managers’ task performance. Therefore,

*Proposition 4*: Technological reflectiveness and individual ambidexterity are personal resources related to digital managerial tasks.

## 5. Discussion

### 5.1. Theoretical implications

This conceptual article has important theoretical implications for organizational behavior and strategic management scholars interested in digital managerial tasks. First, we highlighted the central role of understanding managers’ work engagement in digital managerial tasks. We explain how digital businesses can affect job demands and resources with consequences for task performance. We also argue that managers’ work engagement positively relates to task performance in digital adoption and digital business model tasks.

The framework presented in this paper links digital managerial tasks with job demands and resources using the traditional JD-R model (Bakker and Demerouti, 2017). Hence, companies should be explicitly considered human-centered design principles such as work engagement in developing and procuring new technologies in their digital adoption strategies and the design process of new digital business models (*cf.* Table 1).

We propose that sustained attention, timely reaction, and maintenance and deployment are job demands for digital managerial tasks. Theoretically, the selection of a new digital task involves job demands that managers should perceive as relevant for training new executives (Gunasekaran et al., 2017). Managers must formulate investment plans to acquire new job resources related to digital managerial tasks, such as BDAI and automation. Otherwise, the imbalance between the new job demands and the lack of new job resources can trigger managers to fall into negative states of mind, such as exhaustion or burnout. Our theoretical framework proposes that companies with digital managerial tasks should facilitate the development of new job and personal resources to make new job demands affordable (Demerouti, 2022). We discussed that technological reflectiveness and individual ambidexterity are personal resources for managers involved in digital managerial tasks (Torres et al., 2015; Andrade-Valbuena and Torres, 2018). Furthermore, companies with digital business models must focus on upskilling and reskilling managers, board members, and other stakeholders to help them adapt to technology shifts (Meijerink et al., 2021). Finally, all these contributions open exciting avenues for further development, like empirical studies that confirm or challenge our propositions.

### 5.2. Practical implications

This paper also contributes to practice. In particular, it emphasizes the importance of managers increasing their level of work engagement. We suggest that companies with digital business models formulate long-term investments to increase BDAI assimilation and automation. Additionally, the chief executive officer (CEO), the top management team (TMT), and middle managers must understand the boundaries of the automation process in their organization and develop and train specific personal resources to make strategic decisions (Torres et al., 2015). New digital demands put pressure on managers to acquire more personal resources continuously. The development of personal resources also presents companies with challenges of how managers overcome cognitive limitations to exploit new resources (Dierickx and Cool, 1989).

Nevertheless, from this cognitive perspective, managers’ mental limitations also affect how they understand and respond to new job demands, but above all, how managers make decisions in dynamic and complex environments (Torres et al., 2017). Companies with digital business models should consider that a misperception of job resources might affect how managers cope with new job demands (e.g., difficulty understanding and perceiving new job resources; Sterman and Dogan, 2015). In such a line, the JD-R model could trace new elements based on which managerial cognition researchers can improve efficiency in the relationship between a firm’s human capital and the digital operations dimension (Tehseen et al., 2021).

Finally, using the JD-R model, our theoretical framework describes several contingency factors that may predict managers’ well-being and task performance. For instance, a company needs to generate managers’ work engagement to perform better. Engaged managers are more creative in promoting an innovative climate for other employees and have better creative performance (Juyumaya and Torres, 2023). Firms need to address these issues *via* diverse strategies, such as talent management, training and development, and incentive programs to handle manager work engagement in the workplace (Asija and Ringov, 2021). Firms must integrate work engagement into the strategy and operations to design new and better job positions in the proposed JD-R framework (*cf.* Figure 1).

### 5.3. Directions for future research

The conceptual framework presented here opens up several exciting avenues for further research. A new venue is adopting a managerial cognitive perspective to explain how managers deal with new job demands. Drawing upon the JD-R model, organizational psychology, organizational behavior and strategic management researchers can discover new biases and decision-making heuristics when managers adopt digital managerial tasks (Tversky and Kahneman, 1974). Besides, the individual’s quest for more job and personal resources is a crucial challenge for further research. Nearby to personal resources, the JD-R model can be expanded to consider personal demands (Bakker and Demerouti, 2017). Future studies could explore how managers face their cognitive limitations (e.g., misperception) to face new job and personal demands.

Due to managers facing more visual attention and timely reaction demands, visual perception play a crucial role. New research can analyze the manager’s visual strategies to understand work engagement and task performance. Scholars can use the lens of the JD-R model to explain shifts in work engagement or one of the work engagement dimensions, such as absorption. Owing to the nature of visual perception, researchers can use neuroscience methods (e.g., eye-tracking data) to develop a multifaceted investigation about managers’ work engagement linking the new job demands and resources presented in this paper.

Another essential direction is the empirical test of our four research propositions using scales that can capture new job demands and resources. On the other hand, qualitative researchers can collect individuals’ perceptions to build new definitions of the job demands, job resources, and personal resources presented in this research. For instance, managers’ work engagement stories could be collected to analyze the degree of technological reflectiveness and individual ambidexterity on managers.

## 6. Conclusion

Since its inception, the JD-R model has inspired hundreds of studies and papers to predict positive and negative consequences of job resources and job demands on employees’ states of mind in the workplace. We further develop and understand managers’ work engagement in the context of digital managerial tasks and complement the traditional JD-R model by studying managers’ work engagement. Introducing new job demands and resources helps managers, scholars, researchers, and policymakers understand the manager’s psychological consequences of implementing digital business models. In this line, this work aims to inspire research to increase the opportunities to build manager well-being and achieve organizational functioning considering the challenging digital world of the following years of the XXI century. We hope this research contributes to developing new theoretical and empirical studies about managers’ work engagement and digital managerial tasks.

## Author contributions

JJ supervised, conceptualized, visualized, edited, and wrote the article. JT conceptualized, visualized, edited, and wrote the article. All authors contributed to the article and approved the submitted version.

## Funding

This article was supported by the Agencia Nacional de Investigación y Desarrollo (ANID) under grant Beca de Doctorado Nacional 21190010 and grant Fondecyt de Iniciación 11190146.

## Conflict of interest

The authors declare that the research was conducted in the absence of any commercial or financial relationships that could be construed as a potential conflict of interest.

## Publisher’s note

All claims expressed in this article are solely those of the authors and do not necessarily represent those of their affiliated organizations, or those of the publisher, the editors and the reviewers. Any product that may be evaluated in this article, or claim that may be made by its manufacturer, is not guaranteed or endorsed by the publisher.
